# Safety, Tolerability, and Dose-Limiting Toxicity of Lacosamide in Patients With Painful Chronic Pancreatitis: Protocol for a Phase 1 Clinical Trial to Determine Safety and Identify Side Effects

**DOI:** 10.2196/50513

**Published:** 2024-03-07

**Authors:** Evan L Fogel, Jeffrey J Easler, Ying Yuan, Dhiraj Yadav, Darwin L Conwell, Santhi Swaroop Vege, Samuel Y Han, Walter Park, Vanessa Patrick, Fletcher A White

**Affiliations:** 1 Department of Medicine School of Medicine Indiana University Indianapolis, IN United States; 2 Department of Biostatistics The University of Texas MD Anderson Cancer Center Houston, TX United States; 3 Department of Medicine University of Pittsburgh School of Medicine Pittsburgh, PA United States; 4 Department of Internal Medicine Mayo Clinic Rochester, MN United States; 5 Department of Medicine The Ohio State University Wexner Medical Center Columbus, OH United States; 6 Department of Medicine Stanford University Medical Center Stanford, CA United States; 7 Department of Anesthesia School of Medicine Indiana University Indianapolis, IN United States

**Keywords:** abdominal pain, opioid-induced hyperalgesia, pain, abdomen, protocol, toxicity, toxic, lacosamide, pancreas, pancreatitis, opioid

## Abstract

**Background:**

Chronic abdominal pain is the hallmark symptom of chronic pancreatitis (CP), with 50% to 80% of patients seeking medical attention for pain control. Although several management options are available, outcomes are often disappointing, and opioids remain a mainstay of therapy. Opioid-induced hyperalgesia is a phenomenon resulting in dose escalation, which may occur partly because of the effects of opioids on voltage-gated sodium channels associated with pain. Preclinical observations demonstrate that the combination of an opioid and the antiseizure drug lacosamide diminishes opioid-induced hyperalgesia and improves pain control.

**Objective:**

In this phase 1 trial, we aim to determine the safety, tolerability, and dose-limiting toxicity of adding lacosamide to opioids for the treatment of painful CP and assess the feasibility of performance of a pilot study of adding lacosamide to opioid therapy in patients with CP. As an exploratory aim, we will assess the efficacy of adding lacosamide to opioid therapy in patients with painful CP.

**Methods:**

Using the Bayesian optimal interval design, we will conduct a dose-escalation trial of adding lacosamide to opioid therapy in patients with painful CP enrolled in cohorts of size 3. The initial dose will be 50 mg taken orally twice a day, followed by incremental increases to a maximum dose of 400 mg/day, with lacosamide administered for 7 days at each dose level. Adverse events will be documented according to Common Terminology Criteria for Adverse Events (version 5.0).

**Results:**

As of December 2023, we have currently enrolled 6 participants. The minimum number of participants to be enrolled is 12 with a maximum of 24. We expect to publish the results by March 2025.

**Conclusions:**

This trial will test the feasibility of the study design and provide reassurance regarding the tolerability and safety of opioids in treating painful CP. It is anticipated that lacosamide will prove to be safe and well tolerated, supporting a subsequent phase 2 trial assessing the efficacy of lacosamide+opioid therapy in patients with painful CP, and that lacosamide combined with opiates will lower the opioid dose necessary for pain relief and improve the safety profile of opioid use in treating painful CP.

**Trial Registration:**

Clinicaltrials.gov NCT05603702; https://clinicaltrials.gov/study/NCT05603702

**International Registered Report Identifier (IRRID):**

PRR1-10.2196/50513

## Introduction

### Background

Chronic pancreatitis (CP) is a syndrome characterized by inflammation and progressive architectural destruction of the pancreas. This may result in pancreatic fibrosis with concomitant loss of exocrine and endocrine function, regardless of etiology. The most common symptom of CP is debilitating abdominal pain, which may be constant or flare intermittently. The health care and socioeconomic consequences of CP may be substantial. Notably, painful disease exacerbations often prompt medical evaluation. Patients with constant pain often require daily analgesics for pain relief; further, they may experience disability, subsequent loss of productivity, and frequent need for hospitalization [1].

The treatment of abdominal pain is idiosyncratic and typically based on the skill set of the provider or the resources available at an individual institution. The management of pain in patients with CP has traditionally been based on the assumption that all symptoms have a structural basis. However, in a recent review, it was noted that central sensitization and psychological and social factors are now recognized to potentially contribute to chronic pain, supporting the expansion of therapeutic approaches to include nonstructural behavioral interventions [2]. Although the authors recommended avoidance of chronic opioid use in this patient population, opioids remain a mainstay of therapy at many centers in the United States. However, opioid use may present serious risks, including overdose and opioid use disorder. From 1999 to 2014, >165,000 people died of overdose related to opioid pain medication in the United States [3]. In addition, opioid monotherapy is often insufficient for the management of chronic pain of any etiology. Notably, the single-agent maximum-tolerated dose (MTD) of these drugs may reduce pain by only 25% to 40%, owing to incomplete efficacy, dose-limiting adverse effects, or both. The side effects of opioids, including marked detrimental effects on respiratory, gastrointestinal, and cardiovascular functions, may limit the use of opioid analgesia. In 2016, the Centers for Disease Control and Prevention published a guideline for prescribing opioids for chronic pain, noting that nonpharmacological and nonopioid pharmacological therapies are preferred for chronic pain [4]. If opioids are used, they should be combined with nonpharmacological therapy and nonopioid pharmacological therapy, if possible. Therefore, to minimize side effects, clinicians should prescribe the lowest effective opioid dose. Successful development of an opioid-minimizing strategy would provide clinicians with a new tool to lower the prescribed opioid doses*.* Studies addressing the efficacy of alternative methods for pain management are necessary. We propose an innovative study and intervention that focuses on the safety and tolerability of a novel combination of narcotic and nonnarcotic approach to pain experienced by patients with CP.

Adjuvant drugs, including antidepressants and anticonvulsants, are often used in combination with opiates for the treatment of pain with modest outcomes. One rather pronounced adverse off-target effect of opioids is an increasing sensitivity to noxious stimuli, even evolving a painful response to previously nonnoxious stimuli (allodynia). This condition is clinically known as opioid-induced hyperalgesia (OIH) [5,6]. The underlying pathophysiology of OIH is complex and may be mediated by central [7-9] or peripheral [10] pathways. An intervention targeted against 1 or more of these mechanisms may potentially limit OIH and lead to improved pain control in patients requiring opioids. On the basis of preclinical published data [11], preliminary studies presented subsequently, and clinical observations [12], we hypothesize that therapeutic targeting of the sodium channel NaV1.7 will improve opioid efficacy for controlling pain in patients with CP. Voltage-gated sodium channels (NaVs) play a key role in the initiation and propagation of action potentials in electrically excitable nociceptive neurons. Specific NaVs in these neurons can be activated by nonclassical opioid receptors [11,13]. This activation and augmentation of NaV-dependent pain sensing appears to counter a few classical opioid effects [14-16]. Therefore, neuronal activation of NaV1.7 could be one of the mechanisms that limit opioid effectiveness [11,13,15,16]. The activation of these channels can be blocked with an existing class of Food and Drug Administration (FDA)–approved antiepileptic seizure drugs that modulate NaVs [11,17-20]. The recent identification of neurobiological mechanisms associated with NaV1.7 and opioid efficacy provide the innovative rationale for this combinatorial opioid adjuvant therapy. The basic drug mechanism is that nonclassical opioid receptors function to increase the NaV1.7 current in nociceptive sensory neurons, leading to drug-induced pain, or OIH. The combination of an opioid and specific antiseizure drugs such as carbamazepine, oxcarbazepine, or lacosamide effectively diminishes the opioid-induced NaV1.7 current [11,13,15]. These preclinical observations in several neuropathic pain-induced injury models lay the foundation for changing the management of chronic pain from empirical symptom control to personalized targeting of specific mechanisms responsible for pain.

Lacosamide is a first-in-class FDA-approved modified amino acid antiepileptic drug that targets NaV1.7. It was approved by the FDA after phase 3 trials as adjunctive and monotherapy in adults with refractory partial epileptic seizures [21]. The mechanism of action of lacosamide is thought to be that it enhances the slow inactivation of NaV1.7 without affecting its fast inactivation [17-20]. Lower concentrations of this drug may also bind to fast-inactivated states in a manner similar to other antiseizure agents but with slower kinetics of binding and unbinding [20]. Therefore, this means that lacosamide only affects neurons that are depolarized or active for long periods, which is typical of neurons not only at the focus of epilepsy but also central to active pain states. We chose lacosamide as our study drug over other sodium channel inhibitors because it has a favorable side effect profile. Carbamazepine use as an adjunct to opioids is limited by its adverse effects; therefore, the reduced side effect profile of lacosamide may make it a better choice for chronic pain management [22]. In addition, carbamazepine is a well-known inducer of hepatic drug metabolism and therefore may result in a variety of drug interactions that might compromise the efficacy and safety of ongoing therapies. Lacosamide, on the other hand, has minimal interaction with hepatic cytochrome P450 drug-metabolizing enzymes [23]. Lacosamide is a *CYP2C19 substrate* [24]. The drug has a negligible first-pass effect, with a bioavailability of approximately 100%. The maximum lacosamide plasma concentrations occur approximately 1 to 4 hours after oral administration, and the pharmacokinetics of lacosamide are dose proportional. Food intake does not affect its absorption. Notably, *CYP2D6* is responsible for the metabolism of most of the commonly prescribed opiate medications, including codeine, tramadol, hydrocodone, and oxycodone [25]. Furthermore, the *CYP3A4* system regulates the excretion of fentanyl, methadone, and buprenorphine. However, patients receiving methadone will be excluded from participation in Safety, Tolerability, and Dose-Limiting Toxicity of Lacosamide in Patients With Painful Chronic Pancreatitis (STTEPP), as this is a μ-opioid agonist and acts as an antagonist of the N-methyl-D-aspartate (NMDA) receptor [26].

Several studies have evaluated lacosamide as a monotherapy for clinical pain conditions such as fibromyalgia and painful diabetic peripheral neuropathy. Although all studies claimed that lacosamide reduced neuropathic pain and was well tolerated, the outcomes were generally inconclusive and provided only marginal benefits [27-31]. Notably, however, all these studies excluded patients who required opioids for pain control. One clinical case report suggested that chemotherapy-induced painful peripheral neuropathy uncontrolled by opioids alone is accompanied by a dramatic reduction in pain when lacosamide was added to opioid therapy [12]. This provides clinical evidence supporting our rationale for the use of lacosamide in combination with opioids to treat neuron-based symptoms. Therefore, we propose to evaluate lacosamide in conjunction with opioids, which is where evidence consistently shows a benefit. Evidence regarding the effect of lacosamide on NMDA receptor has been shown by its ability to prevent seizures and death in NMDA-induced convulsion test paradigms in mice [32].

### Objectives

In this protocol, we will evaluate the safety, tolerability, and dose-limiting toxicity (DLT) of the combination of lacosamide and opioid therapy in patients with CP, as well as the feasibility of the performance of this phase 1 pilot study. The MTD will be determined. Lacosamide, an anticonvulsant that is safe and well tolerated in patients with epileptic seizures, may potentially lower the opioid dose necessary for adequate pain relief in patients with CP. The positive outcomes of this pilot study will then support proceeding with a phase 2 efficacy trial assessing the ability of opioid adjuvants to alleviate abdominal pain in this difficult patient population. In addition, we believe that this combination therapy will serve to significantly enhance the safety profile of opioid use in these patients.

## Methods

### Study Organization

The Consortium for the Study of Chronic Pancreatitis, Diabetes, and Pancreatic Cancer (CPDPC) has been tasked by the National Institutes of Health (NIH) with the comprehensive clinical, epidemiological, and biological characterization of patients with CP to develop treatments and gain insight into the pathophysiology of CP and its sequelae, including chronic pain, pancreatic exocrine and endocrine insufficiency, and diabetes pancreatic cancer association [33]. All participating clinical centers in the STTEPP trial are CPDPC members and are contributing to a longitudinal cohort study called *Prospective Evaluation of Chronic Pancreatitis for Epidemiologic and Translational Studies* (PROCEED) [34], the first prospective cohort study of pancreatitis in the United States. STTEPP was funded by the National Institute of Diabetes and Digestive and Kidney Diseases (NIDDK; R01DK132709) in April 2022 as a multi-institutional prospective ancillary study within the CPDPC. MD Anderson Cancer Center, University of Texas, will serve as the data coordinating center for STTEPP, and the Institutional Review Board (IRB) at MD Anderson will serve as the single-center IRB for this study. The goal of this clinical research study is to determine whether adding the antiseizure medication lacosamide to opioid pain medications will help in managing chronic, ongoing abdominal pain symptoms caused by CP. The safety of this drug combination will also be studied. This is an investigational study.

### Lacosamide

Lacosamide is a Schedule V controlled substance approved by the FDA for the management of seizure disorders. A controlled substance is a drug or other substance that is tightly controlled by the government, because it may be abused or cause addiction. All controlled substances in the United States are classified into 5 schedules. Schedule V drugs, including lacosamide, have the lowest potential for misuse and addiction compared with those in other schedules. Although it has been used off-label to treat pain resulting from nervous system damage, this medication is not FDA approved for the management of abdominal pain. Currently, it is being used to treat abdominal pain for research only. There may or may not be benefits for participants in this study. The participant will be closely followed by a study coordinator and site investigator during participation. Although the actual drug therapy will only last for 7 days, the participant may experience improved control of their abdominal pain symptoms during the study. Future patients may also benefit from what is learned in this study.

### Ethical Considerations

This study protocol was approved by the University of Texas MD Anderson Cancer Center Institutional Review Board (IRB ID 2021-1197). Informed consent was obtained from all participants or their legal guardians. All methods were performed in accordance with relevant guidelines and regulations.

Participation is completely voluntary. Before choosing to participate in this study, participants should discuss any concerns they may have with the study team, including side effects, potential expenses, and time commitment. The participant can read the full list of potential side effects in the *Possible Risks* section of the consent form.

The participant may choose not to take part in this study, and they do not have to participate. They may withdraw from the study at any time. The participant has the right to choose whether to participate in this research study. They may decide to stop participating in the study once they have started. Leaving the study early will not result in any loss of benefits that they already have. Instead of participating in this study, they may choose to continue with their current medications and plan of care.

### Study Details

#### Who Is Eligible to Take Part in the Study?

Patients with ongoing chronic abdominal pain related to CP, even with opioid pain medication treatment, were asked to participate in the study.

#### How Many Participants Are Expected to Take Part in the Study?

Approximately 24 participants will participate in this multicenter study from approximately 5 institutions. Up to 10 participants will be enrolled at Indiana University.

#### How Long Will the Participation Be? How Long Will It Take to Complete the Study?

If the patient decides to take part in this study, they will complete a screening visit in person to determine if they qualify for enrollment. This visit will take approximately 30 to 60 minutes. After the screening visit, participation will involve an in-person visit that will last 60 minutes. The participant will be asked to take the study medication (provided at no cost) for the 7 days of participating in the study. The participant will also keep a medication diary to confirm when and how they take the study medications and their pain medications. The final study visit will be an in-person follow-up visit on day 8, lasting approximately 60 minutes. The study team will collect medical history and clinical information, draw blood samples, and ask the patient to complete the visit questionnaires as well as medication diaries of all pain medications they take, including the study medication. The research data will be maintained indefinitely.

#### What Will Be Done to the Patient If They Agree To Be a Research Subject?

If a patient wants to participate in the study, they will be asked to sign the consent form and Health Insurance Portability and Accountability Act (HIPAA) authorization before starting any study procedures. If they agree to participate in the study, the study team will collect several items on the days of their study visits.

### Screening Visit

The screening visit will be completed in person and is expected to last 30 to 60 minutes. The protocol for the screening visit has been described in Textbox 1.

**Protocol for screening visit.**

- The participant will be asked about the medicines they have used or are now taking, including all prescriptions and nonprescription medications.
- They will also be asked to provide demographic information (eg, age and ethnicity).
- They will have blood (approximately 7 teaspoons) collected for routine safety tests. These blood tests are important to determine whether they are eligible to participate in the study and to monitor for possible side effects of the study drug. They should fast (ie, eat nothing and drink only water) for at least 8 hours before this blood investigation.
- If the patient can become pregnant, they will undergo a urine pregnancy test. To participate in this study, they must not be pregnant.
- The patient will have an electrocardiogram test performed to measure the electrical function of their heart.
- They will be asked to complete 4 questionnaires related to pain and how it affects their daily life.
- They will be given a medication diary to track the medications they take for pain.
- The study team will review their medical records to confirm eligibility to continue in the study.

### Enrollment Visit

The enrollment visit will be completed in person on day 0 and is expected to last for 60 minutes. The protocol for enrollment visit day and the treatment days 1 to 7 is described in Textbox 2.

**Protocol for enrollment visit and treatment days.**

**Enrollment visit day 0**

- The patient will be asked if they have any health problems other than chronic pancreatitis and what medical or surgical procedures they have undergone in the past.
- They will be asked to complete 4 questionnaires related to their pain and how it affects their daily life.
- They will be asked about any health problems they may be experiencing.
- They will be asked what medications they are taking, including all prescription and nonprescription medications.
- Patients’ vital signs (eg, heart rate, blood pressure, and temperature), height, and weight will be recorded.
- They will have a physical examination.
- If they can become pregnant, they will have a urine pregnancy test.
- Patients will be given a 7-day supply of the study drug and medication diaries to document all medications taken for pain.

**Treatment days 1 to 7**

- The lacosamide tablets can be stored at room temperature. Patients will take lacosamide by mouth, 2 times a day, approximately 12 hours apart. They will be asked to try and take the tablets at the same time on each of the 7 days of the study. The tablets may be consumed with or without food. Patients will be asked to swallow the tablets whole with a glass of water or any other liquid of their choice and not chew them.
- Patients will be asked to complete daily medication diaries for all medications taken for pain, including the study drug.

### Follow-Up Visit

The first follow-up visit will be completed in person on day 8 and is expected to last for 60 minutes. Subsequently, a follow-up visit will be conducted via telephone on day 21. The protocol for these follow-up visits in described in Textbox 3.

**Protocol for follow-up visits.**

**Follow-up visit on day 8**

- Patients will be asked to complete 4 questionnaires related to pain and how it affects their daily life.
- They will be asked about any health problems they may be experiencing.
- They will be asked what medications they have taken, including all prescriptions and nonprescription medications from the enrollment visit day 0.
- Their vital signs will be recorded.
- They will have a physical examination.
- Blood (approximately 7 teaspoons) will be collected for routine safety investigations. They will need to fast before the blood is collected.
- They will have an electrocardiogram test performed to measure the electrical function of their heart.
- They will return any leftover study drug doses to this visit, in the original bottles.

**Follow-up on day 21: phone visit**

- Patients will be asked about any health problems they may be experiencing.
- They will be asked what medications they have taken, including all prescriptions and nonprescription medications, from the follow-up visit on day 8.

### Risks Associated With the Study

#### Risks of Receiving the Study Drug

There are risks to participating in any research study. One risk is that the study drug does not relieve the abdominal pain. There is a risk of side effects or feeling bad during this study. Side effects vary from person to person. Everyone participating in this study will be carefully monitored for side effects. It is important that the patients tell the study physician about any side effects that they have during this study, even if they do not think it is related to the study drug. The side effects may range from mild to severe. Study physicians and the company that makes the study drug cannot know all the side effects that may occur, and there may be unknown side effects.

#### Lacosamide Side Effects

Lacosamide intake can result in common, occasional, or rare but serious side effects.

- *Common* side effects (occurring in >20% of the patients):
  - Dizziness

- *Occasional* side effects (occurring in 3%-20% of the patients):
  - Headache, fatigue, sleepiness, skin wound, nausea, vomiting, diarrhea, bruising, tremors, loss of coordination, difficulty in walking, double vision, blurry vision, eye twitching, and injection site pain

- *Rare but serious* side effects (occurring in fewer than 3% of patients):
  - Irregular or slow heartbeat, fainting, fever, difficulty in forming words or speaking, abnormal brain function (eg, affecting balance and coordination), mental status change (eg, memory loss and impaired thinking), suicidal thoughts or behavior, hallucinations (ie, seeing or hearing things that are absent), liver damage because inflammation, abnormal sensation (eg, pins and needles), walking or balance problems (eg, possible falling), and life-threatening allergic reactions (eg, difficulty breathing, low blood pressure, and organ failure)

- Lacosamide may rarely cause low blood cell counts (red or white blood cells).
  - A low red blood cell count (anemia) may cause difficulty in breathing or fatigue. The patient may require a blood transfusion.
  - A low white blood cell count increases the risk of infection (eg, pneumonia or severe blood infection). Infections may occur anywhere and become life-threatening. Symptoms of infection may include fever, pain, redness, and difficulty in breathing.

In other studies of lacosamide, stopping the drug suddenly did not lead to unpleasant physical symptoms. As a Schedule V controlled substance, lacosamide has a very low potential for abuse. No restrictions on activities need to be followed when taking this drug. However, should the patient have a side effect or feel bad during this study, they should call the study physician. The study physician’s telephone number is on the front page of the consent form. They should also call the physician if they have a side effect that makes them visit the hospital during the study.

#### Other Risks

Collecting blood for investigations may cause pain, bleeding, and bruising. The patient may faint and develop an infection with redness and irritation of the vein at the site of blood collection. Frequent blood collection may cause anemia (low red blood cell count), which may necessitate blood transfusions. Undergoing an electrocardiogram (EKG) may cause discomfort while lying on the examination table, and the tape on the EKG pads may cause skin irritation.

The questionnaires may contain questions that are sensitive in nature. They may refuse to answer any question that may make them feel uncomfortable. If the patient has concerns about completing the questionnaire, they are encouraged to contact their physician or the study chair.

The arm cuff used in measuring blood pressure may feel tight or slightly uncomfortable for a short time, and during the measurement, their hand or arm may experience numbness.

#### Certificate of Confidentiality

This study is covered by a Certificate of Confidentiality (CoC) from the NIH. With this CoC, the researchers may not disclose or use information that may identify the patient in any federal, state, or local civil; criminal; administrative; legislative; or other action, suit, or proceeding or be used as evidence, for example, if there is a court subpoena, unless the patient has consented for this use. Information protected by this CoC cannot be disclosed to anyone else who is not connected with the research, except if there is a federal, state, or local law that requires disclosure (eg, reporting child abuse or communicable diseases but not for federal, state, or local civil; criminal; administrative; legislative; or other proceedings).

The CoC cannot be used to refuse a request for information, for auditing or program evaluation purposes, from personnel of the United States federal or state government agencies sponsoring the project. A CoC does not prevent the patient from voluntarily releasing information about themselves or their involvement in this research. If the patient wants their research information to be released to an insurer, medical care provider, or any other person not connected to the research, they must provide consent to allow the researchers to release it. The CoC will not be used to prevent disclosure for any purpose you have consented.

#### Pregnancy-Related Risks

Taking part in this study can result in risks to an unborn or breastfeeding baby, so the patient should not become pregnant, breastfeed a baby, or father a child while in this study. They must use birth control during the study if they are sexually active.

- Male patients should tell the physician right away if their partner becomes pregnant or suspects pregnancy.
- If female patients are pregnant, they will not be enrolled in this study. If they become pregnant or suspect pregnancy, they must tell their physician immediately. Pregnancy will result in exclusion from this study.

#### Risk of Possible Loss of Confidentiality

Every effort will be made to keep patients’ personal information confidential; however, we cannot guarantee absolute confidentiality. No information that could identify the patient will be shared in the publication of this study. Patients’ personal information may be shared outside the research study if required by law and with individuals or organizations that oversee the conduct of research studies, and these individuals or organizations may not be held to the same legal privacy standards as physicians and hospitals.

There are some types of sharing the CoC does not apply to. The CoC does not stop reporting required by federal, state, or local laws, such as reporting of child or elder abuse, some communicable diseases, and threats to harm oneself or others. The CoC does not stop a government agency who is funding research from checking records or evaluating programs. The CoC also does not prevent their information from being used for other research when allowed by federal regulations. The CoC also does not stop sharing of information required by the FDA.

Researchers may release information about the patient when they say that it is okay. For example, the patient may still give them permission to release information to insurers, medical providers, or others not connected to the research.

### Costs and Compensation

#### Will Participants or Their Insurers Be Billed for Any Costs of the Study? If so, Which and What Happens if Insurance Does Not Cover the Costs?

The study will pay for the study drug, blood collection, ECGs, physical examinations, and data collection associated with the research study and for the investigations that are performed for research purposes only.

#### What Happens if the Patient is Hurt or Became Sick as a Result of the Study?

The researchers have taken steps to minimize the known or expected risks. However, they may still experience problems or side effects, even when the researchers are cautious to avoid them. If the patient believes that they have been harmed, they should notify the researchers listed in Section 6 of the consent form. The team at Indiana University Hospital will provide first aid or emergency care. The cost of this first aid or emergency care may be billed to the patient’s insurance company. Additional medical care will be provided if the injury is determined to be caused by the research. If they sign the form, they do not give up their right to seek additional compensation if they are harmed because of being in this study.

Of note, it is important that the patients tell the researchers about any injuries, side effects, or other problems that they experience during this study. They may also need to tell their regular physicians. Furthermore, it is the patient’s responsibility to determine the extent of their health care coverage. There are no other monetary compensation programs in place for such injuries. They will not be reimbursed for expenses or compensated financially by MD Anderson or the NIH and NIDDK for this injury. They may also contact the Chair of MD Anderson’s IRB at 713-792-6477 with questions about study-related injuries.

#### Are Patients Paid or Given Anything for Being in the Study?

The patient will be paid US $50 at the completion of enrollment visit day 0 and US $100 at the completion of follow-up visit day 8; both payments will be in the form of gift cards.

### Patient Eligibility

Relevant to STTEPP, PROCEED study participants are divided into subcohorts to distinguish the natural history of different disease presentations at the time of admission into the cohort [34]. In STTEPP, we will leverage the resources of the CPDPC by approaching participants already enrolled in PROCEED. This approach will facilitate timely accrual, as these participants have demonstrated an interest in research and willingness to participate in CPDPC studies. Although all participants must meet the criteria for diagnosis of suspected or definite CP as per PROCEED definitions [34], study enrollment will not be limited to participants participating in PROCEED. The STTEPP inclusion and exclusion criteria are listed in Textboxes 4 and 5, respectively.

**Inclusion criteria.**

1. Written informed consent and Health Insurance Portability and Accountability Act (HIPAA) authorization for release of personal health information
2. Age ≥18 years at the time of informed consent
3. Suspected (YELLOW 2 or 3) or definite (RED) diagnosis of chronic pancreatitis, as per the Consortium for the Study of Chronic Pancreatitis, Diabetes, and Pancreatic Cancer’s *Prospective Evaluation of Chronic Pancreatitis for Epidemiologic and Translational Studies* (PROCEED) study definition with ongoing symptoms of abdominal pain
4. Patients must be maintained on an opioid (except methadone or suboxone) for 4 weeks before enrollment for the treatment of abdominal pain related to pancreatitis, with a daily morphine equivalent dose of 20 mg to 120 mg.
5. Ongoing symptoms of abdominal pain even with opioid use (Visual Analog Score and Brief Pain Inventory average score ≥4, at enrollment)
6. Eastern Cooperative Oncology Group performance status of 0 to 2 (Oken et al [35])
7. Ability to swallow and tolerate oral tablets
8. Females of childbearing potential must have a negative pregnancy test
9. The following laboratory parameters must be met: white blood cell count ≥3.0 K/mm^3^, absolute neutrophil count ≥1.5 K/mm^3^, hemoglobin ≥9 g/100 ml, platelets ≥75 K/mm^3^, creatinine ≤1.5 mg/100 mL, bilirubin ≤1.5 x upper limit of normal (ULN), aspartate transaminase ≤3 ULN, alanine transaminase ≤3 ULN; normal PR interval on baseline 12-lead electrocardiogram.

**Exclusion criteria.**

1. Participants with indeterminate chronic pancreatitis (YELLOW 1) as per *Prospective Evaluation of Chronic Pancreatitis for Epidemiologic and Translational Studies* (PROCEED) criteria
2. Treatment with any investigational agent within 30 days before registration or concurrent participation in a clinical trial which involves another investigational agent
3. Rapidly escalating pain that requires parenteral (eg, intravenous or intramuscular) opioid therapy within 30 days of enrollment
4. Known hypersensitivity or allergic reaction to lacosamide, carbamazepine, or oxcarbazepine
5. Pregnant or breastfeeding
6. Diagnosis of epilepsy or a patient who is currently taking antiepileptic drugs
7. Abdominal surgery or pain intervention (eg, endoscopic retrograde cholangiopancreatography with sphincterotomy or stent or stone removal and celiac plexus block) within 90 days of enrollment.
8. Hospitalization for pancreatitis exacerbation or pain management within 90 days of enrollment
9. Patient who currently takes Suboxone or methadone.
10. Other factors which might explain the patient’s ongoing symptoms, at the discretion of the enrolling physician.
11. History of autoimmune or traumatic pancreatitis or sentinel attack of acute necrotizing pancreatitis which results in suspected disconnected duct syndrome.
12. Primary pancreatic tumors, pancreatic ductal adenocarcinoma, suspected cystic neoplasm (>1 cm in size or main duct involvement), neuroendocrine tumors, and other uncommon tumors
13. Pancreatic metastasis from other malignancies
14. History of solid organ transplant and HIV or AIDS
15. Known isolated pancreatic exocrine insufficiency (eg, without any eligible inclusion criteria)
16. No medical or psychiatric illnesses or ongoing substance abuse that, in the investigator’s opinion, would compromise participant’s ability to tolerate study interventions or participate in follow-up.

### Objectives and End Point

Our primary objectives are to (1) evaluate the safety, tolerability, and DLT of lacosamide in combination with opioids in patients with CP and (2) assess the feasibility of the performance of a pilot study of adding lacosamide to opioid therapy in patients with CP. As a secondary, exploratory objective, we will assess the efficacy of adding lacosamide to opioid therapy for the treatment of abdominal pain because of CP. The safety end point includes toxicity and DLT of the combination of lacosamide and opioids, defined as grade 3 or 4 toxicities, according to the National Cancer Institute Common Terminology Criteria for Adverse Events v5.0. Tolerability will be assessed by compliance with the intervention. The participants will be evaluated for completing the 7-day trial. The percentage of participants taking 100%, 75%, 50%, and <50% of the study tablets will be recorded. The feasibility end point will include the recruitment rate (ie, the proportion of eligible patients who agree to participate) and the dropout rate, including a qualitative assessment of barriers to retention. The efficacy end point will be assessed using the Visual Analog Score; Brief Pain Inventory, short form, average score; and the Comprehensive Pain Assessment Tool, short form [36] total score. A 50% decrease in pain scores from baseline at study completion will be deemed clinically meaningful. In addition, opioid use will be evaluated, with a decrease of 25% in pain scores from baseline at study completion, identified as clinically significant.

### Investigational Plan, Intervention, and Treatment Regimen

**Figure 1:**
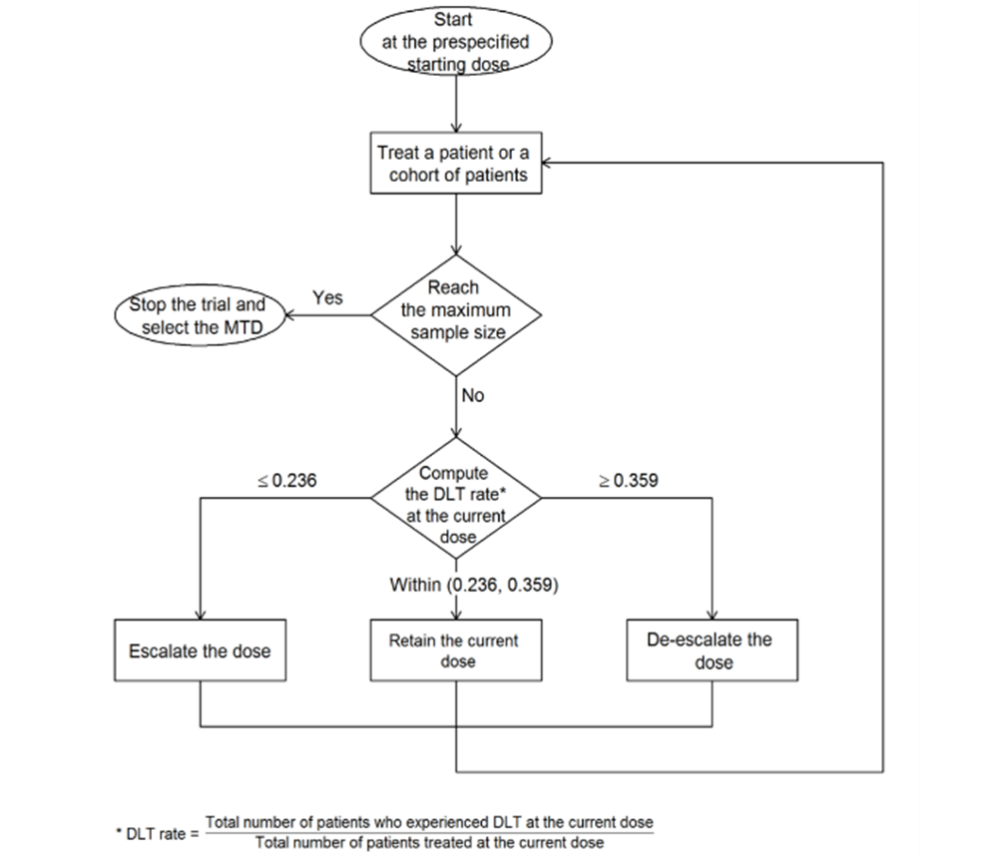
Bayesian optimal interval (BOIN) schema. The BOIN schema is a model-assisted dose-finding design that can be used to determine the maximum-tolerated dose (MTD) of a study drug based on safety or the optimal biological dose (OBD) based on safety and efficacy. DLT: dose-limiting toxicity.

We will conduct a dose-escalation trial of lacosamide added to opioid therapy in patients with chronic abdominal pain owing to CP. Participants who meet all the inclusion criteria and none of the exclusion criteria will be included in this dose-finding study. We will use the Bayesian optimal interval (BOIN) [37,38] design to determine the MTD. The BOIN design received a fit-for-purpose designation from the FDA as a drug development tool [39]. The BOIN design is implemented in a simple manner similar to the traditional 3+3 design, but it is more flexible and possesses superior operating characteristics compared to those with more complex model-based designs, such as the continual reassessment method [40]. The target DLT rate for MTD is 0.3, and the maximum sample size is 24. We will enroll and treat patients in cohorts of size 3. The initial dose will be 50 mg taken orally twice a day (100 mg/day), followed by an incremental increase of 100 mg/day in 2 divided doses. The maximum daily dose of lacosamide will be 400 mg/day. The duration of lacosamide administration will be 7 days at each dose level. This target DLT rate and dose-escalation schedule were chosen based on previous trials that had suggested the efficacy and tolerability of comparable doses of lacosamide when used to treat partial seizures and painful diabetic neuropathy. Follow-up laboratory parameters (as obtained at study entry) will be obtained on day 8 (with a 3-day window) after therapy is completed. The trial design is illustrated in Figure 1 and is described as follows:

1. Patients in the first cohort are treated at dose level 1.
2. To assign a dose to the next cohort of patients, dose escalation or de-escalation will be performed according to the rules displayed in Table 1.
3. Repeat step 2 until the maximum sample size of 24 is reached or stop the trial if the number of patients treated at the current dose reaches 15.

In the BOIN design, the number of DLT is the number of patients with at least 1 DLT. When none of the actions (ie, escalation, de-escalation, or elimination) is triggered, the current dose remains unchanged for the next cohort of patients. “N/A” means that a dose cannot be eliminated before treating 3 evaluable patients. After the trial is completed, we will select the MTD based on isotonic regression [38]. This computation is implemented by the “Estimate MTD” tab of the BOIN Design Desktop Program [41]. Notably, each cohort of 3 patients will consist of 3 new patients. Therefore, dose escalation or de-escalation is interpatient (or, more precisely, intercohort), not intrapatient in nature.

Considering the low disease prevalence of CP, including referral bias at academic centers, we chose to avoid the use of eligibility criteria that would narrow the potential recruitment pool. In this phase 1 trial, in which safety, tolerability, and feasibility are the primary outcomes of interest, we propose including all patients with suspected or definitive CP if the above inclusion and exclusion criteria are met. Similarly, although there may be potential variability in patients because of the etiology, duration of CP, or other features of the disease, it is impractical to impose more stringent eligibility criteria during this early phase of the investigation. Doing so would substantially deplete the recruitment pool for this orphan disease and would not be feasible or scientifically justified.

**Table 1:** Dose-escalation or de-escalation rule for the Bayesian optimal interval design.

Actions	The number of patients treated at the current dose														
	1	2	3	4	5	6	7	8	9	10	11	12	13	14	15
Escalate if the number of DLT^a^ is ≤	0	0	0	0	1	1	1	1	2	2	2	2	3	3	3
De-escalate if the number of DLT is ≥	1	1	2	2	2	3	3	3	4	4	4	5	5	6	6
Eliminate if the number of DLT is ≥	N/A^b^	N/A	3	3	4	4	5	5	5	6	6	7	7	8	8

^a^DLT: dose-limiting toxicity.

^b^N/A: not applicable.

### Drug Dispensing and Compliance

In this phase 1 trial, all enrolled participants will receive the active drug lacosamide. There is no placebo group, and there is no blinding to the treatment or dose received. Following the initial drug purchase, the investigational drug service at each participating institution will purchase additional disbursements on an as-needed basis, depending on patient recruitment. Participants will be provided with drug diaries, which will be reviewed for compliance at the end of the study at the final in-person visit. Patients who miss a dose (defined as beyond 2 hours of the patient’s typical drug administration time) will not be instructed to take it later on the same day. Patients will be informed not to take missed doses on the subsequent day (ie, the patient should not take more than the prescribed daily dose).

### Study Visits

A screening visit for eligibility in person or via telephone will occur at 7 to 30 days prior to enrollment. There will be 2 in-person study visits. During study visit 1 on day 0, baseline study assessments and questionnaires will be completed in person. Drug treatment days will then occur from days 1 to 7. During study visit 2 on day 8 (with a 3-day window), following completion of the 7-day drug treatment period, participants will have a face-to-face clinic visit, where similar assessments and questionnaires will be completed, as per the study calendar (Table 2). Participants will return all unused drugs during this visit for disposal and to monitor compliance.

**Table 2:** Study calendar.

		Screening visit, from 7 to 30 days	Enrollment visit, day 0	Treatment days 1-7	Follow-up visit, day 8 (+3-day window)
**Required assessments**					
	Informed consent	✓			
	Medical history^a^		✓		
	Height and weight		✓		
	Physical examination		✓		✓
	Vital signs		✓		✓
	ECOG^b^ performance status	✓	✓		✓
	EKG^c^	✓			✓
	Blood chemistries^d^	✓			✓
	Hemoglobin, absolute neutrophil count, and platelets	✓			✓
	Urine pregnancy	✓	✓		
	Medication assessment^e^	✓	✓		✓
	Adverse event assessment		✓		✓
**Questionnaires**					
	Physician questionnaire		✓		
	Study coordinator questionnaire		✓		✓
	Patient questionnaire (VAS^f^, BPI^g^, PROMIS^h^, and COMPAT^i^)	✓	✓		✓
	Medication diary	✓	✓		✓

^a^Medical history and establishment of suspected or definite chronic pancreatitis diagnosis may occur at any time before trial entry.

^b^ECOG: Eastern Cooperative Oncology Group.

^c^EKG: electrocardiogram.

^d^Will include at a minimum: electrolytes, glucose, blood urea nitrogen, creatinine and creatinine clearance, calcium, and hepatic function panel.

^e^Will include detailed use of rescue pain medications and opioids use.

^f^VAS: Visual Analog Score.

^g^BPI: Brief Pain Inventory.

^h^PROMIS: Patient-Reported Outcomes Measurement Information System.

^i^COMPAT: Comprehensive Pain Assessment Tool for Chronic Pancreatitis.

### Study Measurements

A site gastroenterologist will review the complete medical history, including medications (particularly opioid use), Eastern Cooperative Oncology Group performance status, physical examination (including vital signs, height, and weight), EKG, and laboratory test results. A site research nurse will collect up to 35 mL of fasting blood using standard protocols. Given the safety or risk protocol of lacosamide and to satisfy inclusion and exclusion criteria, the blood specimens are considered routine safety laboratories and will include electrolytes, glucose, blood urea nitrogen, creatinine, creatinine clearance, calcium, and a hepatic function panel.

The patient, nurse coordinator, and physician case report forms (CRFs) will be completed before study entry. This will be based on a similar CRF currently used in PROCEED. Completion of these forms before enrollment will allow confirmation that the patient is eligible for the study. We will obtain approval from the single-center IRB to share data between protocols with the patients’ consent. An additional CRF will be completed by the nurse coordinator in collaboration with the site-specific principal investigator at the follow-up study visit, documenting all side effects noted during the drug treatment phase according to Common Terminology Criteria for Adverse Events (version 5.0).

### Safety or Risk Issues

Lacosamide is a Schedule V medication available in tablet, oral solution, and injectable forms. In this study, only the tablet form will be used, and this is available in 50 mg, 100 mg, 150 mg, and 200 mg dosages. For simplicity in drug ordering and distribution, only 50 mg tablets will be used. Lacosamide is FDA approved as monotherapy or adjunctive therapy for partial-onset seizures, with a maximal recommended oral total daily dose of 400 mg, administered in 2 divided doses.

### Summary of Known Potential Risks With Study Medication

#### Side Effects

A complete list of reported side effects from lacosamide trials and postmarketing experience can be found in the medication insert and Amneal Pharmaceuticals website [42]. The common side effects of lacosamide at doses in the range of 200 mg to 400 mg include dizziness (25%), ataxia (6%), fatigue, nausea, diplopia, headache, or tremor (12%-17%). Severe side effects such as cardiac rhythm or conduction abnormalities (first-degree arteriovenous block and atrial fibrillation or flutter, 0.5%) and suicidal ideation or behavior (0.2%) are rare. Case reports have been published regarding episodes of syncope and hypersensitivity reactions.

#### Prior and Concomitant Medications or Procedures

Relevant information about all concomitant drugs (including prescribed, over-the-counter, or herbal preparations) taken before and during the trial and any dose or dose regimen changes that occur during the trial will be recorded in the source documents and case report form. Owing to the potential drug-to-drug interaction with other CYP3A4 or CYP2C9 inhibitors that may interfere with lacosamide levels, patients taking medications that may interfere with lacosamide metabolism will be monitored carefully. Furthermore, patients taking medications that affect cardiac conduction (eg, sodium channel blockers, β-blockers, and calcium channel blockers), including those that prolong the PR interval, will be carefully observed. We will obtain an EKG before beginning drug therapy and at the end of treatment**.**

The study principal investigators serve as the medical monitors for this trial, overseeing all aspects of the trial, including management of source documentation, adverse event collection, adverse events and protocol deviation reporting, interaction with the Data and Safety Monitoring Board (DSMB), and the creation of corrective and preventive action plans, when necessary. The DSMB will meet to review the study conduct and data relating to safety and efficacy to ensure the continued scientific validity and merit of the study. Following recruitment of the first study participant, summaries will be submitted monthly and reviewed quarterly by the DSMB and will follow the established CPDPC protocols.

### Statistical Plan and Analysis

A sample size of 24 was determined and calibrated based on the simulation such that the phase 1 trial has reasonable accuracy (>60% probability) to correctly identify the MTD across a set of practically plausible scenarios. We will use descriptive statistics to summarize the demographic and clinical characteristics of the patients. Safety data will be tabulated by the grade and type of toxicity using descriptive statistics, including mean, SD, and 95% CI. The reduction in pain scores will be summarized by the dose using the mean and 95% CI. As an exploratory analysis, *t* test (2-sided) or Wilcoxon rank-sum test will be used to compare the reduction in pain scores between the doses. The recruitment and dropout rates will be calculated for all patients and by dose and used to determine the feasibility of the treatment. Although the data generated will not have a comparator group (ie, no placebo), these will be helpful in understanding the variability of responses in patients with suspected or definite CP, which in turn will be useful for sample size calculation for a follow-up trial.

## Results

As of December 2023, we have currently enrolled 6 participants. The minimum number of participants to be enrolled is 12 with a maximum of 24. We expect to publish the results by March 2025.

## Discussion

The management of abdominal pain in patients with CP remains a significant challenge, and innovative approaches to pain management are urgently needed. By potentially limiting OIH, lacosamide may improve pain control in patients requiring opioids. There are no data evaluating the use of lacosamide in patients with CP; this was the impetus of the STTEPP trial. Achieving recruitment goals is a priority. We acknowledge that patients may be unwilling to participate in a dose-finding trial with a low likelihood of long-term therapeutic benefit. Those patients who do note a benefit, however, may choose to be considered for our planned phase 2 trial. The 5 participating centers include experienced researchers, who have led clinical trials in this challenging patient population. In addition, all are members of the CPDPC and participate in PROCEED [38]. This brings substantial synergy to this study because the infrastructure for recruitment and follow-up of study participants is already in place at these institutions. We do not require the study entry to be limited to patients enrolled in PROCEED, to avoid limiting the recruitment pool. However, placing our initial focus on patients participating in PROCEED, particularly those who have demonstrated compliance and return for their follow-up visits, will identify motivated patients who are familiar with the study protocols and the importance of research. The short-term nature of this study should facilitate compliance. Because the safety data of combination therapy with lacosamide and opioids are unknown in patients with CP, this phase 1 trial is necessary. We initially considered the conventional 3+3 design for this study protocol, which suffers several limitations, such as low accuracy in identifying and estimating the MTD and the tendency of underdosing patients. Therefore, we adopted a novel phase 1 trial design (ie, the BOIN design) that allows better statistical learning of the dose-toxicity curve and more reliable identification of the MTD [37].

This study will provide new knowledge regarding the safety, toxicity, and DLT of lacosamide in patients with CP. It is anticipated that lacosamide will prove to be safe and well tolerated. The results of this pilot study will then support proceeding with a phase 2 trial of assessing the efficacy of lacosamide added to opioid therapy to alleviate abdominal pain caused by CP. We will demonstrate that these pilot trials, in which long-term therapeutic benefits are unlikely, are feasible in this challenging population. This knowledge will facilitate future clinical trials on CP, providing data regarding patient engagement and recruitment that will better inform trial design. Future clinical trials may indicate that a combination of lacosamide and an opioid will achieve better analgesia at lower doses of each drug than either as a single agent.

## Abbreviations

BOIN: Bayesian optimal interval
CoC: Certificate of Confidentiality
CP: chronic pancreatitis
CPDPC: Consortium for the Study of Chronic Pancreatitis, Diabetes, and Pancreatic Cancer
CRF: case report form
DLT: dose-limiting toxicity
DSMB: Data and Safety Monitoring Board
EKG: electrocardiogram
FDA: Food and Drug Administration
HIPAA: Health Insurance Portability and Accountability Act
IRB: Institutional Review Board
MTD: maximum-tolerated dose
NaVs: voltage-gated sodium channels
NIDDK: National Institute of Diabetes and Digestive and Kidney Diseases
NIH: National Institutes of Health
NMDA: N-methyl-D-aspartate
OIH: opioid-induced hyperalgesia
PROCEED: Prospective Evaluation of Chronic Pancreatitis for Epidemiologic and Translational Studies
STTEPP: Safety, Tolerability, and Dose-Limiting Toxicity of Lacosamide in Patients With Painful Chronic Pancreatitis

## Data Availability

Deidentified patient data may be shared with investigators from other institutions for ancillary studies upon request. Requests should be made to the study principal investigator.
